# In Vitro Biological Evaluation of Titanium Implants Anodized With Aqueous Extract of *Psidium guajava* and Conventional Electrolytes

**DOI:** 10.1155/ijbm/5522753

**Published:** 2026-06-02

**Authors:** Felipe Gustavo Dias, Gabriela Zimmermann Prado Rodrigues, Isadora Schell Frozza, Carlos Henrique Amaro da Silva, Fernando Dal Pont Morisso, Cláudia Trindade Oliveira, Günther Gehlen, Ana Luiza Ziulkoski

**Affiliations:** ^1^ Post Graduation Program in Toxicology and Toxicological Analysis, Feevale University, Novo Hamburgo, Rio Grande do Sul, Brazil; ^2^ Post Graduation Program in Environmental Quality, Feevale University, Novo Hamburgo, Rio Grande do Sul, Brazil; ^3^ Post Graduation Program in Materials Technology and Industrial Process, Feevale University, Novo Hamburgo, Rio Grande do Sul, Brazil

**Keywords:** cytotoxicity, guava, NIH-3T3, osseointegration, Saos-2, titanium anodization

## Abstract

Titanium implants can release ions that damage cells. Anodization creates a protective oxide layer, but conventional acid methods raise environmental and safety concerns. This study evaluates in vitro cytotoxicity and osseointegration with the *Psidium guajava* aqueous leaf extract (PgE) as an alternative anodization electrolyte for titanium plates. For this purpose, 1‐cm^2^ titanium plates were anodized with H_3_PO_4_, a combination of H_3_PO_4_+HF or PgE, and each group was assessed for its impact on cell viability, adhesion, and proliferation in osteosarcoma (Saos‐2) and fibroblast (NIH‐3T3) cells. The plate surfaces were characterized by energy‐dispersive X‐ray spectroscopy (EDS), X‐ray diffraction (PXRD), and contact angle measurements using the sessile drop method, demonstrating greater hydrophilicity in plates anodized with PgE. Cytotoxicity was assessed using both indirect assays (extraction media) and direct assays, where cells were seeded directly onto titanium plates; cell viability was determined by MTT and NRU assays after 24 and 96 h. Osseointegration was analyzed by counting nuclei through DAPI staining and adhesion using scanning electron microscopy. In indirect assays, all samples showed no significant difference compared to the control (ANOVA/Tukey’s; *p* < 0.05), while direct assays demonstrated an average increase in cell viability on all anodized plates. The number of cell nuclei increased on H_3_PO_4_ and PgE plates in both cell lines compared to pure titanium. SEM analysis revealed improved cell morphology and adhesion on surfaces anodized with H_3_PO_4_ or PgE, whereas cells on H_3_PO_4_+HF appeared thinner and less spread, suggesting impaired cellular anchorage. The results show that using PgE as an anodization electrolyte for titanium plates caused no cytotoxicity and enhanced cell proliferation and adhesion, outperforming conventional H_3_PO_4_+HF electrolyte. This makes it a promising and sustainable alternative aligned with green chemistry principles.

## 1. Introduction

Bone tissue regeneration, although inherently effective, can be insufficient in the presence of critical‐sized defects, leading to nonunion of fractures [1]. To overcome this challenge, biomaterials engineering has focused on the development of surfaces and structures capable of stimulating the proliferation and differentiation of osteogenic cells, as well as modulating inflammatory responses that directly impact osseointegration [2]. This process, defined as the stable and functional union between bone tissue and an implantable material, depends on biophysical and biochemical events that can be monitored in *in vitro* systems prior to clinical application [3]. Among the key factors determining the success of this process, the biocompatibility of the materials used stands out. In the case of metallic implants, especially those made of titanium, biocompatibility refers to the material’s ability to perform its therapeutic function while promoting cell adhesion and proliferation, without inducing cytotoxic effects on the host tissue [4].

Despite their widespread use and high resistance, titanium implants may undergo corrosion upon contact with the biological environment. To enhance their durability, surface treatments are applied to thicken the titanium dioxide (TiO_2_) layer, aiming to minimize adverse tissue reactions [5]. Among the surface treatment methods, anodization stands out by rendering the implant surface rougher and more hydrophilic, thereby enhancing cell adhesion and proliferation [6].

However, conventional electrolytes used in anodization, such as phosphoric and hydrofluoric acids, raise toxicological risks. Phosphoric acid, for instance, has demonstrated moderate toxicity in mice exposed via inhalation [7], whereas hydrofluoric acid has been associated with dermal burns and systemic toxicity in occupational exposure studies [8]. In the search for safer and more sustainable alternatives, plant extracts such as *Psidium guajava* have been proposed as green electrolytes for titanium anodization [9]. The oxidizing effect of *P*. *guajava* leaf extract (PgE) is probably related to its phenolic derivatives. These compounds are adsorbed onto the metal surface and form phenoxy radicals and quinones, which, in the presence of potential or current, act as oxidizing agents during the anodization process [10]. Moreover, studies using PgE have demonstrated efficacy in inhibiting the growth of various types of human tumor cells, such as glioblastomas, without inducing cytotoxicity in fibroblast cell lines [11].

Therefore, this study compared the interaction and cytotoxicity in two cell models of titanium plates anodized in an electrolyte containing PgE versus conventional acid electrolytes, assessing cell viability, adhesion, and osseointegration with a view to future in vivo application.

## 2. Materials and Methods

### 2.1. Preparation of Electrolytes

For the electrolyte containing only phosphoric acid (H_3_PO_4_), an aqueous solution of H_3_PO_4_ at a concentration of 1 mol/L was used. For the electrolyte composed of phosphoric acid (H_3_PO_4_) and hydrofluoric acid (HF), a 1‐mol/L aqueous solution of H_3_PO_4_ was used with the addition of 0.15% (w/v) HF [12]. For the PgE electrolyte, mature leaves were collected from an urban area in southern Brazil (29.66645° S, 51.11965° W; datum WGS 84), rinsed under tap water, and allowed to drain until completely dry. The leaves were then oven‐dried in a forced‐air circulation chamber (DeLeo) at 45 °C ± 5°C for approximately 72 h. Once fully desiccated, the leaves were shredded in a blender until a fine powder was obtained. For extract preparation, 6 g of this powder was weighed on an analytical balance and mixed with 120 mL of ultrapure water. The suspension was heated to 40°C ± 2°C under constant stirring for 15 min. After that, the solution was allowed to cool to room temperature. Coarse particulates were removed by sieving, followed by vacuum filtration through qualitative grade‐80 filter paper to eliminate finer solids. The final extract volume was 90 mL, with a measured pH of 5.0. Phosphate physicochemical characterization of PgE was performed using Hanna kits, with samples heated in an HI839800 test tube and subsequently analyzed using an HI83300‐01 multiparameter photometer, with results expressed in mg/L.

### 2.2. Anodization of Titanium Plates

Sample preparation was cutting of grade 2 titanium sheets (Titânio Brasil Ltda.) into plates measuring 1 cm × 1 cm × 1 mm (width × height × thickness), which were subsequently divided into four groups for comparative analysis: pure titanium (pure Ti), with no additional treatment; titanium anodized with H_3_PO_4_; titanium anodized with H_3_PO_4_ + HF; and titanium anodized with PgE. For anodization, the plates were initially pickled by etching in a solution of nitric acid (60% v/v) and hydrofluoric acid (40% v/v) for 5 s, rinsed with deionized water, and then dried. The anodization process was performed at a constant voltage of 15 V for 15 s for each plate. The sample was connected as the anode, and titanium was used as the cathode. During the procedure, voltage data were recorded using data acquisition software connected to the power supply. Voltage versus time graphs were automatically generated, allowing for real‐time monitoring of the electrochemical behavior during anodization.

### 2.3. Characterization of the Anodized Layer Surface

The optical (colorimetric), morphological (SEM), spectroscopic (UV–Vis), and electrochemical (cyclic voltammetry) characterization of the titanium surfaces was carried out following the protocol previously established by our research group, and the results are reported in Cerveira et al. (2022). In addition, gold‐sputtered titanium plates were subjected to energy‐dispersive X‐ray spectroscopy (EDS) analyses, performed using a Thermo Scientific UltraDry EDS detector operated with NSS 3.0 software, coupled to a JEOL JSM‐6510LV scanning electron microscope in secondary electron imaging (SEI) mode. Micrographs for EDS mapping were acquired at 1000× magnification and a working distance of 10 mm.

Phase identification and structural characterization were conducted by powder X‐ray diffraction (PXRD) using a Thermo Scientific ARL EQUINOX 1000 X‐ray diffractometer, equipped with a Cu anode X‐ray tube (Cu‐Kα radiation, *λ* = 1.541874 Å) and a curved position‐sensitive detector (CPS), operating at 40 kV and 30 mA, over a 2*θ* range of 20°–100° with a step size of 0.02°. Diffraction patterns were analyzed using Match! software, supported by the Crystallographic Open Database (COD).

Additionally, wettability was evaluated by contact angle measurements using the sessile drop method. An 8.3‐μL droplet was deposited onto the metallic surface at a dosing rate of 1.0 μL/s, and the contact angle was recorded after 5 s of interaction between the droplet and the surface. Simulated body fluid (SBF) was used as the liquid phase. Measurements were carried out using a Labcontrol OCA 15EC goniometer, with data acquisition performed using SCA20_U software (Dataphysics Instruments). The assay was conducted in triplicate at 21.6°C, and measurements were taken at five different points on each sample.

### 2.4. Sterilization of Titanium Plates

Prior to testing, the plates were fully immersed in a 3% sodium lauryl ether sulfate solution for 24 h. Subsequently, they were rinsed with running water and subjected to six consecutive immersion cycles in reverse osmosis–purified water, each lasting 30 min. During these cycles, the items were gently agitated to facilitate the removal of detergent residues. Finally, the items were thoroughly dried and exposed to UVC light (254 nm) for 30 min on each side.

### 2.5. Cell Culture

NIH/3T3 (ATCC CRL‐1658, RRID: CVCL_0594; mouse embryonic fibroblasts) and Saos‐2 (ATCC HTB‐85, RRID: CVCL_0548; human osteosarcoma) cell lines, obtained from the Rio de Janeiro Cell Bank (BCRJ), were cultured in DMEM (Sigma) supplemented with 10% fetal bovine serum (FBS) (Gibco) and maintained at 37°C in a semiopen system with a humidified atmosphere with 5% carbon dioxide (CO_2_).

### 2.6. Cytotoxicity Assay

**FIGURE 1:**
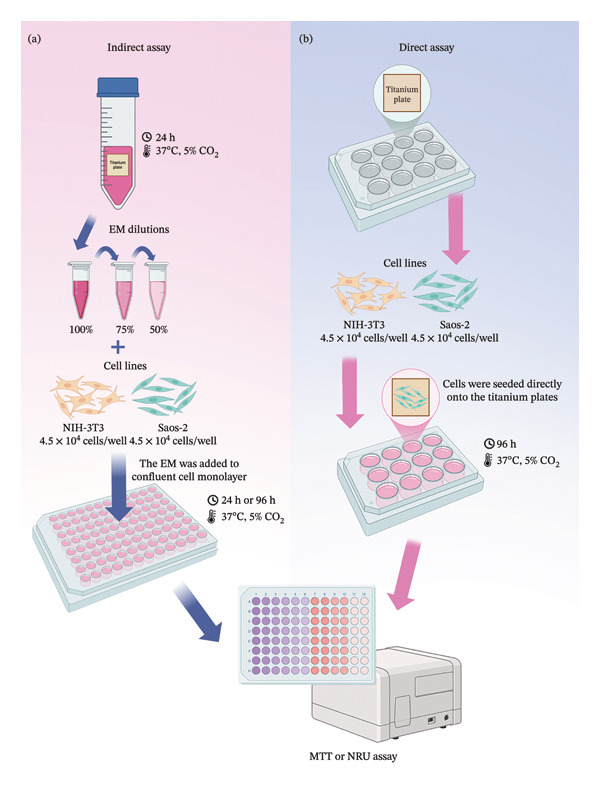
Schematic representation of the experimental design to evaluate the cytotoxicity of anodized titanium plates. (a) Indirect assay: extraction media (EM) were obtained, diluted (100%, 75%, and 50%), and applied to confluent NIH‐3T3 and Saos‐2 cells. (b) Direct‐contact assay: cells were seeded directly onto the surface of titanium plates fixed in 24‐well plates. Cell viability in both methods was assessed after 24 and 96 h using MTT and Neutral Red assays.

For the indirect assay (Figure 1(a)), after sterilization, the titanium plates anodized with different electrolytes were incubated separately in 4 mL of DMEM culture medium per cm^2^ for 24 h at 37°C to obtain the extraction media (EM), containing the soluble fraction released from the plates. Concentrations of 75% and 50% were prepared by diluting the EM in DMEM supplemented with 1% FBS, while the 100% concentration corresponded to the undiluted EM. Meanwhile, NIH‐3T3 fibroblast cells (3 × 10^4^ cells/well) and Saos‐2 osteosarcoma cells (4.5 × 10^4^ cells/well) were seeded in 96‐well plates at 37 °C and 5% CO_2_ for 24 h until confluence. Afterward, the cells were treated with the EM solutions obtained from the titanium plates for 24 h. Untreated cells were used as the negative control, and the cells treated with H_2_O_2_ were used as the positive control. The cells were incubated for 24 h and 96 h. After that, the cell viability was determined by the MTT (Sigma) [12] and Neutral Red (Sigma) uptake (NRU) method [13]. The absorbance was measured in a microplate spectrophotometer SpectraMax M3 (Molecular Devices, USA) at 570 nm and 540 nm, respectively.

For the direct‐contact assay (Figure 1(b)), sterilized titanium plates were fixed into 24‐well plates using 10% carboxymethylcellulose. Cells were seeded directly onto the plates and, after 24 h and 96 h incubation under the same conditions, lysosomal viability was evaluated by the NRU method. Cells in contact with nonanodized titanium plates were used as the negative control. The absorbance was measured in a microplate spectrophotometer at 540 nm.

### 2.7. In Vitro Cell Osseointegration

Osseointegration was analyzed based on proliferation, assessed by DAPI staining for cell nucleus counting, and on cell adhesion, morphology, and interaction with the substrate, evaluated by scanning electron microscopy (SEM).

For 4′,6‐diamidino‐2‐phenylindole (DAPI) staining, NIH‐3T3 fibroblasts and Saos‐2 osteosarcoma cells (1 × 10^4^ cells/well) were cultured in 24‐well plates with titanium plates anodized with different electrolytes for 96 h at 37°C and 5% CO_2_. After the incubation period, the cells were fixed with cold methanol for 10 min. Each titanium plate was then removed from the well and transferred onto a microscope slide. A small drop of Fluoroshield with DAPI (Sigma) was applied to the sample, followed by the careful placement of a coverslip to avoid bubble formation. The samples were observed under a fluorescence microscope Axio Scope. A1 (ZEISS) equipped with a DAPI filter. Twenty images from the corners and the center of each plate were captured using the ZEN software (ZEISS) at 20x magnification. The obtained images were loaded into ImageJ (v.1.53c) in 8‐bit format, and their contrast was adjusted to highlight and isolate the nuclei. Cell nuclei were quantified with a site filter set to 10–500 voxels, excluding edge objects and applying an average threshold value of 43.5. The total number of nuclei counted was divided by the field‐of‐view area and then multiplied by the area of the titanium plates to obtain the total estimated number of nuclei. The results were exported for the calculation of mean values and standard deviations.

For SEM analysis, NIH‐3T3 fibroblast cells and Saos‐2 osteosarcoma cells (1 × 10^4^ cells/well) were cultured in 24‐well plates containing titanium plates anodized with different electrolytes. The cells were incubated for 96 h at 37°C with 5% CO_2_. After incubation, the samples were fixed with 2.5% glutaraldehyde in 0.2 M phosphate buffer (pH 7.4) for 7 days at 8°C. Subsequently, the samples were gently washed with 0.2 M phosphate buffer three times for 30 min each. Then, the samples were dehydrated in a graded ethanol series (10%–100%), dried using a critical point dryer CPD 030 (BalTec), mounted on aluminum stubs, sputter‐coated Q150RS (Quorum) with gold, and examined under SEM to observe cell morphology and features associated with osseointegration. In addition, pore area was quantified from the SEM images using ImageJ software, and the results were exported for the calculation of mean values and standard deviations.

### 2.8. Statistical Analysis

Statistical analysis was performed using the GraphPad Prism 10.0 software. The data normality was assessed by the Shapiro–Wilk test, and *p* < 0.05 was considered significant. For cell viability data, a one‐way ANOVA with Tukey’s post hoc test was performed. MTT and NRU results were expressed as percentage of cell viability based on the negative control.

## 3. Results and Discussion

### 3.1. Titanium Anodization

**FIGURE 2:**
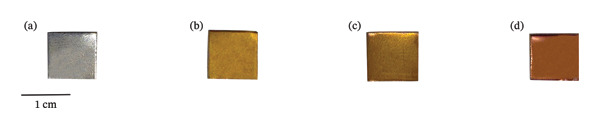
Titanium plates (1 × 1 cm, 1‐mm thick) subjected to different surface treatments: (a) untreated titanium (no anodization); (b) anodized with H_3_PO_4_; (c) anodized with H_3_PO_4_ + HF; (d) anodized using PgE; and (e) pickled titanium. Each color change reflects the influence of the anodization medium on the surface characteristics. Scale bar = 1 cm.

The set of titanium plates, including three anodized using different electrolytic protocols and one nonanodized control, exhibited noticeable differences in color and film homogeneity (Figure 2). While the pure Ti sample retained its characteristic silvery‐gray luster, the plates anodized in H_3_PO_4_, H_3_PO_4_ + HF, and PgE all developed a coppery hue, indicative of successful oxide layer formation.

The electrolyte derived from PgE exhibits a yellowish‐brown color (Figure 2(a)), whereas acid‐based electrolytes are colorless. Therefore, the coloration observed in the anodized samples is not attributed to the original color of the electrolyte, but rather to the changes that occur during the anodization process. The phenomenon of light interference is responsible for the characteristic coloration of the anodized samples, which is associated with the increased thickness of the oxide layer and the variation in applied potential [14], consistent with the colors described in the literature for low applied potentials [15].

**FIGURE 3:**
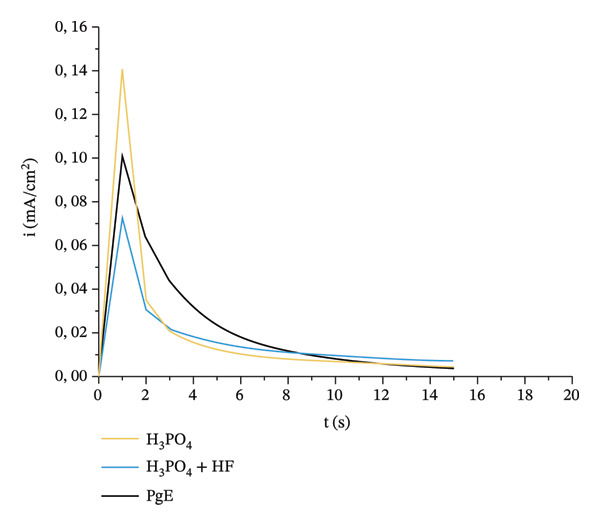
The graph shows current density (mA/cm^2^) versus time (s) during the anodization of titanium plates in three different electrolytes.

Current‐density transients indicate that all samples exhibit an initial peak within the first seconds of anodization, consistent with a charge‐transfer‐controlled process in which species initially adsorbed on the metal surface are consumed (Figure 3) [16]. The largest peak was observed for the H_3_PO_4_, followed by PgE and the H_3_PO_4_+HF. In plates anodized with H_3_PO_4_, the current density reached a maximum of approximately 0.14 mA/cm^2^ at 1.5 s and then declined sharply to below 0.02 mA/cm^2^ by around 6 s. For plates treated with H_3_PO_4_ + HF, the peak current was approximately 0.07 mA/cm^2^ at 1.5 s, followed by a decay to about 0.01 mA/cm^2^ at 6 s. Anodization with PgE produced a peak current of roughly 0.10 mA/cm^2^ at 1.5 s, with a slightly more gradual decay compared to the inorganic electrolytes.

Previous studies have reported that titanium samples anodized in H_3_PO_4_+HF exhibit lower peak currents owing to an HF‐induced oxide formation–dissolution process; this behavior was not observed for samples anodized in H_3_PO_4_ alone, indicating the formation of a barrier oxide [17]. The intermediate current‐density peak observed for PgE highlights the possibility of porous oxide formation. Moreover, the pronounced decay of current density with anodization time for the PgE‐treated samples suggests that part of the measured current continues to be consumed by anodic dissolution during oxide growth, further supporting the likely formation of a porous oxide.

### 3.2. Characterization of the Anodized Layer Surface

EDS analysis (Figure 4(a)) did not detect phosphorus‐bearing phases in all samples, as neither the spectra nor the elemental compositions indicated the presence of such compounds. The unlabeled peak observed at 2.12 keV is attributed to the gold (Au) coating applied during sample preparation. To further investigate the possible presence of phosphorus, particularly in crystalline form, the samples were subjected to PXRD analysis (Figure 4(b)). The obtained diffractograms show that, for all analyzed samples, the diffraction patterns are dominated by the characteristic peaks of metallic titanium with a hexagonal close‐packed crystal structure. The main diffraction peaks are consistent across all samples, confirming the predominance of the titanium substrate.

FIGURE 4Physicochemical characterization of titanium samples. (a) Energy‐dispersive X‐ray spectroscopy (EDS) spectra, confirming the absence of phosphorus‐containing phases in all samples; the peak at 2.12 keV is attributed to the Au coating. (b) Powder X‐ray diffraction (PXRD) patterns, with consistent diffraction peaks across all samples, indicating the predominance of the titanium substrate. (c) Contact angle (*θ*) measurements showing differences in wettability, with samples presenting the lowest contact angle (*θ* = 51.9 ± 0.56°), indicating increased surface hydrophilicity.

**Figure:**
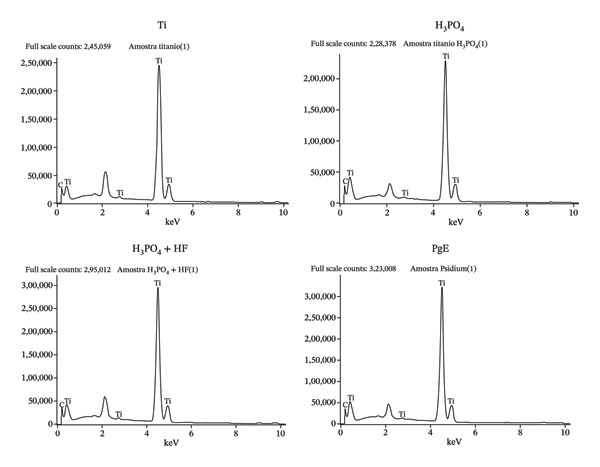
(a)

**Figure:**
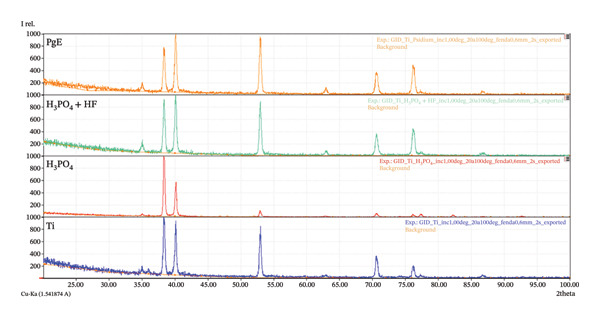
(b)

**Figure:**
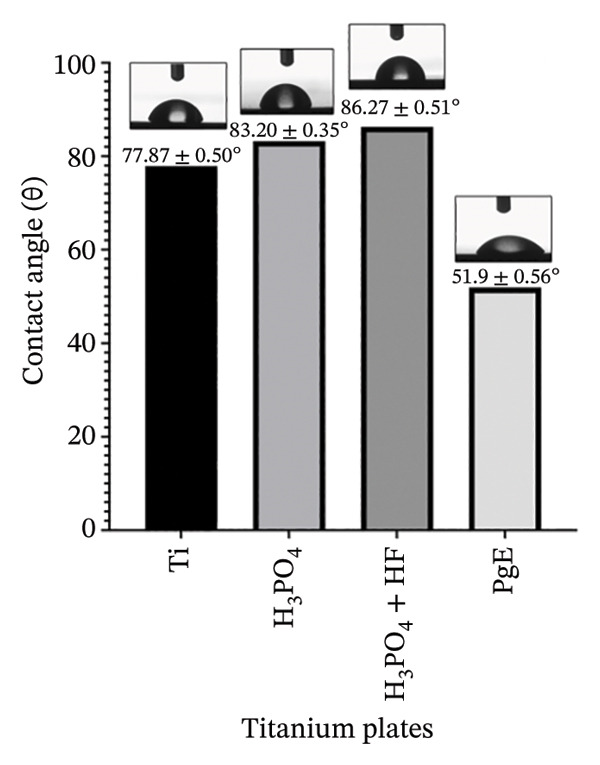
(c)

Taken together, the EDS and PXRD results suggest that the oxide films formed by anodization under the studied conditions are predominantly amorphous or exhibit low crystallinity, which is consistent with the findings reported in the literature [18]. The variations among electrolytes indicate that the anodizing medium affects film structure and its diffractometric response, but no evidence of phosphorus‐containing compounds was observed [19].

Wettability was assessed by measuring the contact angle between a liquid droplet and the surface, as higher surface free energy promotes greater wettability and liquid adhesion [20]. As shown in the contact angle measurements (Figure 4(c)), the Ti sample exhibited a contact angle of 77.87 ± 0.50°, while samples anodized in H_3_PO_4_ (83.20 ± 0.35°) and H_3_PO_4_ + HF (86.27 ± 0.51°) presented higher values. In contrast, the sample anodized in PgE (51.9 ± 0.56°) displayed a more hydrophilic character than the others. These values are within a range that is difficult to categorize in terms of wettability [21]; however, the PgE‐treated surface exhibits comparatively higher hydrophilicity, which may favor cell–surface interactions. Among the crystalline phases that may form during titanium anodization, rutile and anatase, both TiO_2_ polymorphs, are particularly relevant [22]. Rutile is thermodynamically more stable and generally more hydrophobic than anatase [23]. Although the XRD diffractograms did not indicate the presence of either rutile or anatase, the contact angle results suggest a tendency toward rutile‐like behavior for the samples anodized in H_3_PO_4_, with and without HF, and anatase‐like behavior for the sample anodized in PgE. This interpretation is consistent with the formation of a very thin oxide layer.

In the same context, while several studies have reported phosphorus incorporation into anodic oxide films formed on titanium in phosphate‐containing electrolytes [24], neither EDS nor PXRD analyses performed in the present study confirmed such incorporation. This may be attributed to the relatively low phosphate concentration detected in the PgE electrolyte (189.5 ± 35.15 mg/L), combined with the formation of an extremely thin oxide layer on the titanium surface. Notably, previous work from our research group has shown that titanium surfaces anodized in H_3_PO_4_ and PgE exhibit comparable roughness values, suggesting that PgE can reproduce surface characteristics typically associated with phosphoric acid–based anodization [25].

**FIGURE 5:**
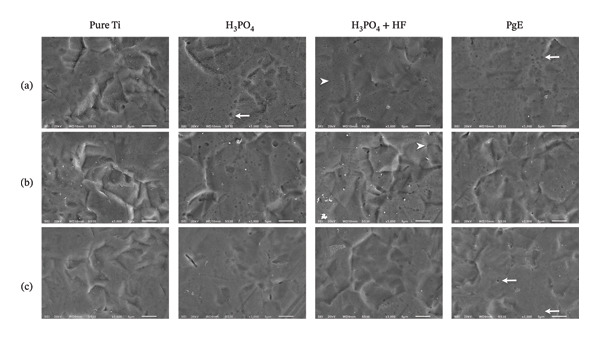
Scanning electron microscopy (SEM) micrographs of titanium plates, either uncoated (pure Ti) or anodized with different electrolytes: H_3_PO_4_, H_3_PO_4_ + HF, and PgE. (a) Surfaces after anodization; (b) surfaces after exposure to DMEM high‐glucose culture medium; (c) surfaces after exposure to DMEM/F12 culture medium. Straight arrows indicate macropores, while arrowheads indicate micropores. Mean pore area: PgE (0.62 ± 0.20 μm^2^), H_3_PO_4_ (1.1 ± 1.15 μm^2^), and H_3_PO_4_ + HF (1.7 ± 0.9 μm^2^). Magnification: 3000×; scale bar = 5 μm.

The SEM analysis revealed distinct surface morphologies among the anodized titanium plates. Images show the surfaces after anodization (Figure 5(a)) and after exposure of the previously anodized samples to DMEM high glucose (Figure 5(b)) and DMEM/F12 (Figure 5(c)) culture media.

Samples treated with the H_3_PO_4_ and H_3_PO_4_ + HF anodization groups exhibited more heterogeneous pores at the micrometric scale, with the H_3_PO_4_ + HF group showing a larger mean area (1.7 ± 0.9 μm^2^) compared to the H_3_PO_4_ group (1.1 ± 1.15 μm^2^) in the present SEM images. In contrast, samples treated with PgE exhibited a smaller and more homogeneous porous structure, with a mean pore area of 0.62 ± 0.20 μm^2^, suggesting that the electrolyte promoted surface modifications. The presence of pores is particularly relevant for biomedical applications, as it has been associated with increased surface area and improved cell–material interactions, favoring osseointegration processes [26]. Although pores were present in all groups, the PgE group showed smaller and more homogeneous pores, considering both the mean pore area and the lower standard deviation. Consistent with this, previous studies have shown that smaller and homogeneously distributed pores on porous titanium surfaces promote early‐stage cell adhesion [27].

In the samples exposed only to culture medium with 1% FBS (DMEM/F12 or DMEM high glucose), pores were present; however, their visualization was more difficult in the SEM images. This observation likely results from the masking effect of adsorbed proteins (from FBS), salts, and/or other medium constituents deposited on the titanium surface during incubation [28]. Altogether, these results suggest that PgE can promote surface porosity comparable to that of conventional anodization methods.

The hypothesis is that the anodization process using PgE is primarily driven by the presence of phenolic compounds composed of carbon, hydrogen, and oxygen in *P. guajava* leaves. These compounds promote oxidation through the interaction of hydroxyl (–OH) species from the electrolyte with the titanium surface, resulting in the formation of a titanium oxide layer [9]. Furthermore, the presence of phosphate may be a factor in the anodization process, since it is found in plant cells. During photosynthesis, solar energy captured by pigments in the photosystems within chloroplasts excites electrons in chlorophyll, which are transferred through a chain of acceptors to regenerate ATP and NADPH via the phosphorylation of ADP [29].

### 3.3. Cytotoxicity

In indirect MTT assays conducted to evaluate the cytotoxicity of titanium plates anodized with different electrolytes (100% concentration), mitochondrial activity exceeded 90% in both cell lines and at both incubation times, indicating absence of toxicity for all anodized plates (Figure 6). No significant differences were observed among the groups, including the control. In Saos‐2 cells, a trend toward increased viability was observed in the H_3_PO_4_ group (141.9%), followed by H_3_PO_4_ + HF (133.6%), pure titanium (126.0%), and PgE (130.0%). After 96 h, viabilities returned to values closer to the control.

FIGURE 6MTT assay evaluating cell viability (%) of Saos‐2 cells at 24 h (a) and 96 h (b), and NIH‐3T3 cells at 24 h (c) and 96 h (d), after exposure to extraction media from titanium plates anodized with different electrolytes at concentrations of 100%, 75%, and 50%. The inset graph in each panel represents the response at 100% extract concentration only. The dotted line at 100% indicates the negative control. Data are expressed as mean ± standard deviation.

**Figure:**
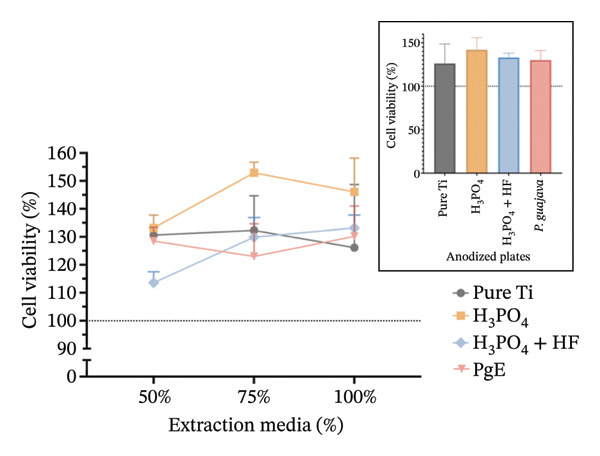
(a)

**Figure:**
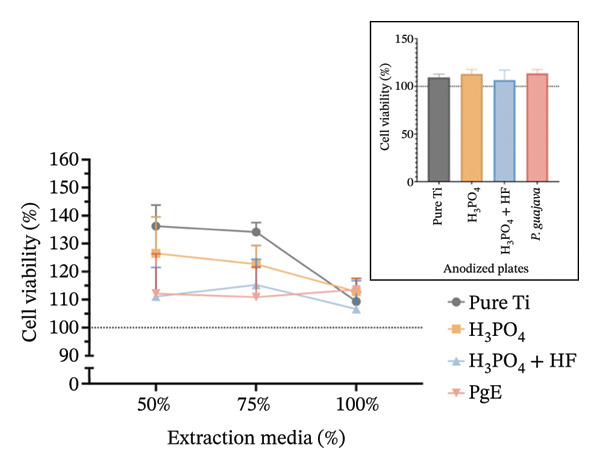
(b)

**Figure:**
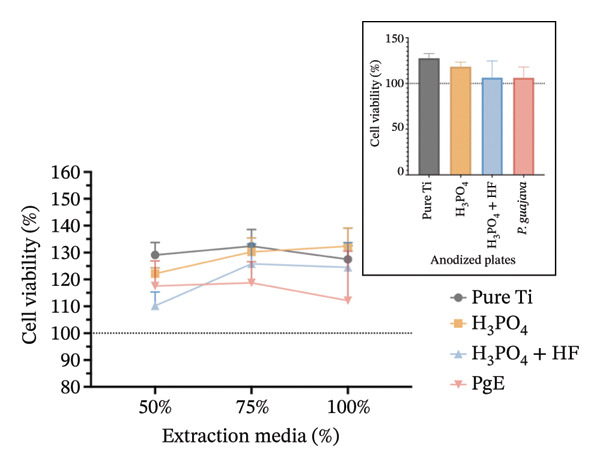
(c)

**Figure:**
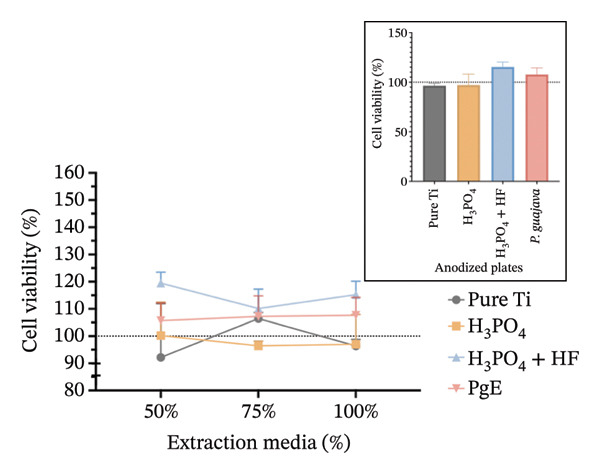
(d)

In indirect NRU assays (Figure 7) conducted under the same conditions, cell viability remained above 100% for all groups. In Saos2 osteoblast‐like cells after 24 h, the H_3_PO_4_‐treated plates elicited the highest uptake (115.8%), followed by *PgE* (106.2%), pure titanium (104.8%), and H_3_PO_4_ + HF (101.8%). At 96 h, *PgE* anodized plates showed the greatest viability (112.3%), with pure titanium next (108.4%), then H_3_PO_4_ + HF (103.0%) and H_3_PO_4_ alone (98.0%). In NIH‐3T3 fibroblasts, NRU at 24 h was highest for H_3_PO_4_ (119.6%), followed closely by H_3_PO_4_ + HF (118.6%), *PgE* (113.9%), and pure titanium (107.1%). After 96 h, the H_3_PO_4_ group again led (112.5%), with *PgE* at 109.9%, H_3_PO_4_ + HF at 104.4%, and pure titanium at 98.0%.

FIGURE 7Neutral red uptake assay evaluating cell viability (%) of Saos‐2 cells at 24 h (a) and 96 h (b), and NIH‐3T3 cells at 24 h (c) and 96 h (d), after exposure to extraction media from titanium plates anodized with different electrolytes at concentrations of 100%, 75%, and 50%. The inset graph in each panel represents the response at 100% extract concentration only. The dotted line at 100% indicates the negative control. Data are expressed as mean ± standard deviation.

**Figure:**
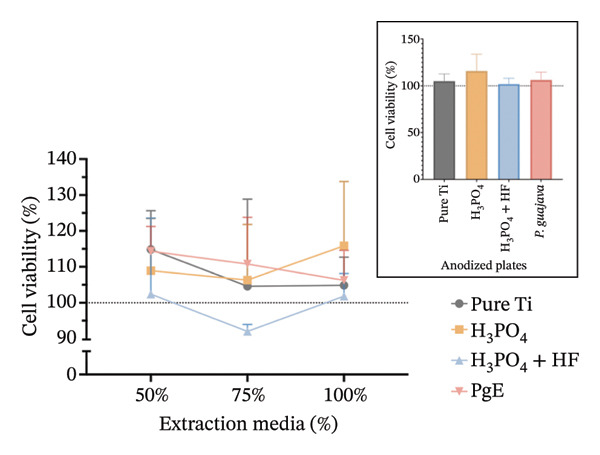
(a)

**Figure:**
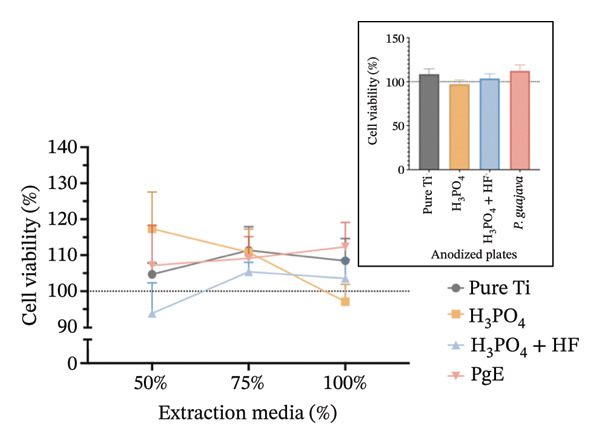
(b)

**Figure:**
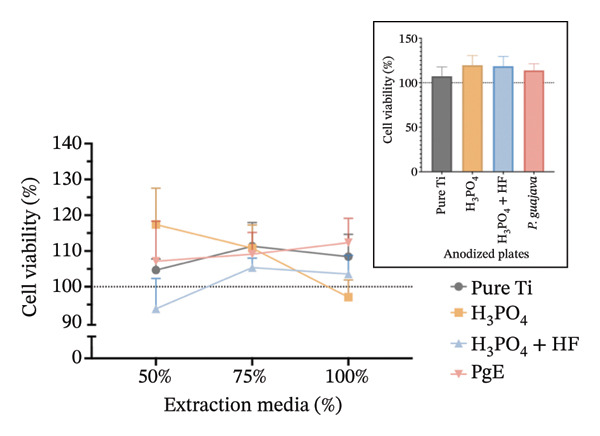
(c)

**Figure:**
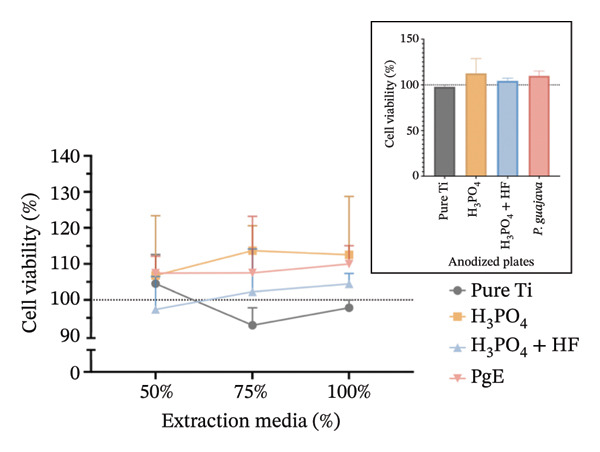
(d)

The results indicate that the anodized plates did not compromise cell viability, meeting the ISO 109935 standards, which classifies materials yielding cell viability of 70% or higher as noncytotoxic [30]. These findings align with the existing literature on the biocompatibility of titanium and its derivatives. Previous studies employing the MTT assay to evaluate the cytotoxicity of various titanium surface treatments have reported that these treatments do not impair cell viability [31]. Similarly, titanium surfaces modified with bioactive coatings did not exhibit cytotoxicity in fibroblasts after 7 days of exposure, with viability remaining above 100% under all tested conditions [32]. The tendency for increased cell viability observed in MTT assay at 24 h should be interpreted with caution, as mitochondrial activity may temporarily increase under stress as an adaptive response. This metabolic surge can elevate absorbance readings without necessarily reflecting a genuine increase in cell proliferation [33]. In the 96‐h assay, however, values normalize and approach the 100% negative control, indicating the cessation of this possible transient effect.

**FIGURE 8:**
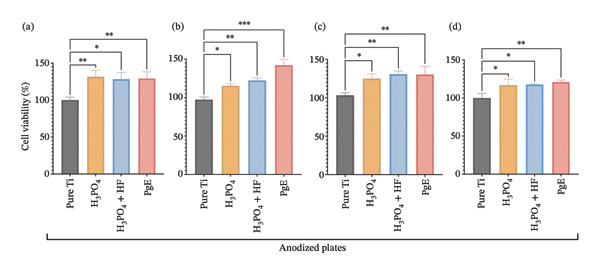
Neutral red uptake assay of cells directly exposed to titanium plates treated with different electrolytes. (a) Saos‐2 at 24 h; (b) Saos‐2 at 96 h; (c) NIH‐3T3 at 24 h; (d) NIH‐3T3 at 96 h. Asterisks indicate statistically significant differences compared to untreated titanium. Data are expressed as mean ± standard deviation. A one‐way ANOVA with Tukey’s post hoc test was performed to compare group means (^∗^ *p* < 0.05, ^∗∗^ *p* < 0.01, ^∗∗∗^ *p* < 0.001).

In direct‐contact assays (Figure 8), none of the titanium plates anodized with H_3_PO_4_ (125%), H_3_PO_4_ + HF (130%), or PgE (130%) induced cytotoxicity in Saos2 cells at 24 h, whereas pure titanium controls showed 103% viability. After 96 h, Saos2 viability remained elevated on H_3_PO_4_ anodized plates (116%), H_3_PO_4_ + HF anodized plates (117%), and PgE anodized plates (120%), compared to 99% on pure titanium. Similarly, NIH‐3T3 cells exhibited increased viability at 24 h on H_3_PO_4_ (126%), H_3_PO_4_ + HF (128%), and PgE (129%) versus 100% on pure titanium, and at 96 h on H_3_PO_4_ (115%), H_3_PO_4_ + HF (122%), and PgE (142%) versus 97% on pure titanium plate.

Direct‐method analyses demonstrated that all tested anodization approaches yielded an average 25.7% increase in cell viability relative to pure titanium. A prior study using human keratinocytes reported that titanium coatings enhance surface roughness and porosity, thereby upregulating the expression of proteins involved in cell adhesion and cell–matrix interactions [34]. In a study evaluating the proliferative effect of mesenchymal stem cells (BMSCs) and human umbilical vein endothelial cells (HUVECs) on pure titanium plates and on plates treated to increase surface roughness, the results revealed significant differences compared to pure titanium [35]. These results demonstrated that physicochemical modifications of the titanium surface, such as those induced by plant‐based electrolytes, can create a more favorable environment for cell proliferation.

### 3.4. Cell Adhesion and Proliferation

**FIGURE 9:**
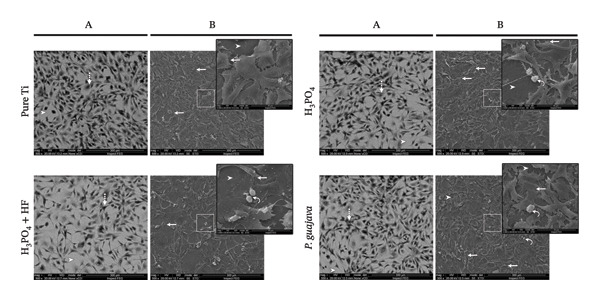
Scanning electron microscopy (SEM) images of NIH‐3T3 cells cultured on titanium plates subjected to different anodization protocols. Column (a) presents images acquired using the backscattered electron detector (vCD mode) at 500× magnification, while column (b) shows images obtained with the Everhart–Thornley detector (ETD mode) at 500×, with a selected region shown at higher magnification (3000×). Arrowheads indicate titanium plates; dashed arrows highlight cells visualized through the vCD filter; straight arrows indicate cells extending cytoplasmic protrusions and extensions to anchor onto the titanium plate; and curved arrows indicate cells undergoing division.

SEM images reveal distinct morphological and proliferative behaviors between the two cell types. NIH‐3T3 fibroblasts (Figure 9) exhibited an elongated and elastic‐like morphology, forming cytoplasmic extensions and displaying incomplete surface coverage, characteristic of fibroblastic cells. On surfaces anodized with H_3_PO_4_ + HF, NIH‐3T3 cells appeared notably thinner than usual, suggesting a potential alteration in cell spreading or adhesion. In contrast, samples anodized with H_3_PO_4_ alone or with PgE showed similar cell morphology and proliferation patterns, indicating favorable surface compatibility for fibroblast growth.

**FIGURE 10:**
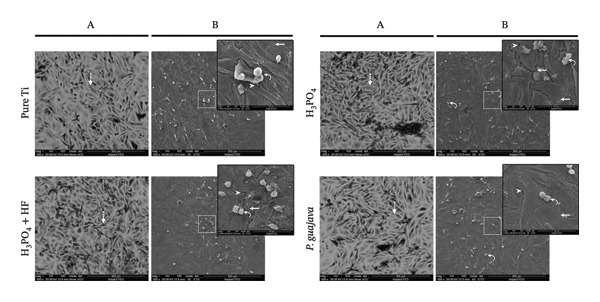
Scanning electron microscopy (SEM) images of Saos‐2 cells cultured on titanium plates subjected to different anodization protocols. Column (a) presents images acquired using the backscattered electron detector (vCD mode) at 500× magnification, while column (b) shows images obtained with the Everhart–Thornley detector (ETD mode) at 500×, with a selected region shown at higher magnification (3000×). Arrowheads indicate titanium plates; dashed arrows highlight cells visualized through the vCD filter; straight arrows indicate cells extending cytoplasmic protrusions and extensions to anchor onto the titanium plate; and curved arrows indicate cells undergoing division.

Saos‐2 cells (Figure 10) exhibited a uniform growth pattern, spreading consistently across the titanium surfaces with limited cytoplasmic extension. On the surfaces anodized with PgE, the cells demonstrated tight intercellular contact and a smooth, well‐spread morphology, effectively covering the substrate. In contrast, cells grown on untreated or acid‐anodized surfaces displayed irregular membrane protrusions and wider spacing between adjacent cells, exposing areas of the substrate, visible as dark background regions in the SEM images.

After 96 h of exposure, DAPI staining was used to visualize and quantify cell nuclei on titanium plates (Figure 11). All anodized plates increased the number of nuclei compared to pure titanium in NIH‐3T3 cells. H_3_PO_4_ treatment resulted in an 84.4% increase in the number of nuclei compared to pure titanium, while H_3_PO_4_ + HF led to a 64% increase. In turn, the PgE electrolyte induced a 99.7% increase.

FIGURE 11DAPI staining and quantification of nuclei from NIH‐3T3 and Saos‐2 cells directly exposed to titanium plates treated with different electrolytes for 96 h. Panel (a) shows fluorescence microscopy images at 10× magnification of DAPI‐stained nuclei; the white scale bar represents 5 μm. In the first column: (A1) NIH‐3T3 on pure Ti, (A2) on H_3_PO_4_, (A3) on H_3_PO_4_ + HF, and (A4) on PgE anodized plates; images (A5–A8) correspond to Saos‐2 cells on the same sequence of anodized plates. Panel (b) presents the estimated number of NIH‐3T3 nuclei on the three anodized titanium surfaces and nonanodized plate, and panel (c) shows the corresponding analysis for Saos‐2 cells. Data are expressed as mean ± standard deviation. A one‐way ANOVA with Tukey’s post hoc test was performed to compare group means (^∗^ *p* < 0.05, ^∗∗^ *p* < 0.01, ^∗∗∗^ *p* < 0.001).

**Figure:**
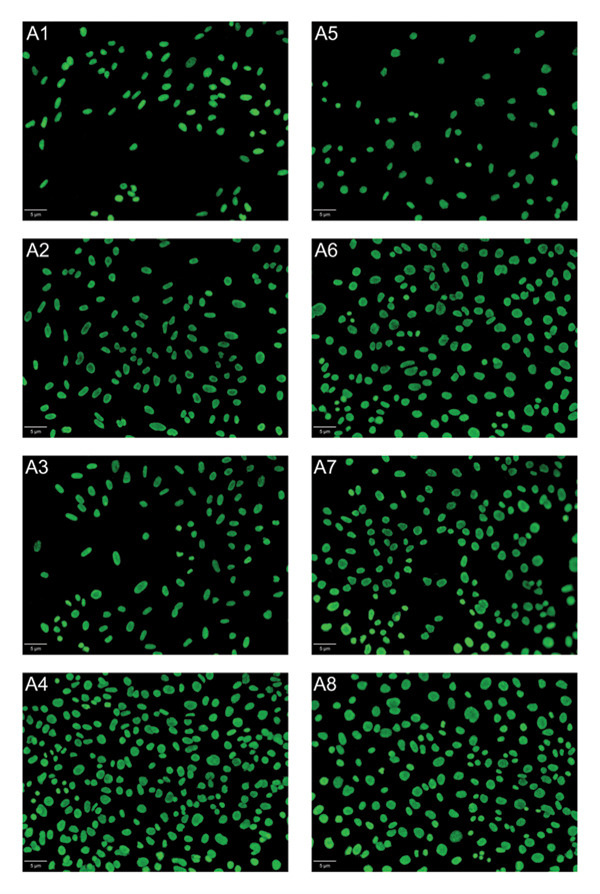
(a)

**Figure:**
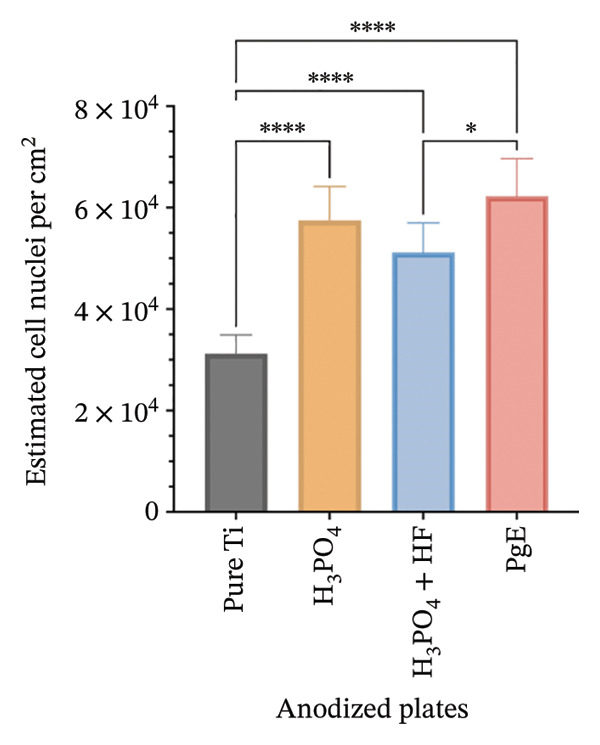
(b)

**Figure:**
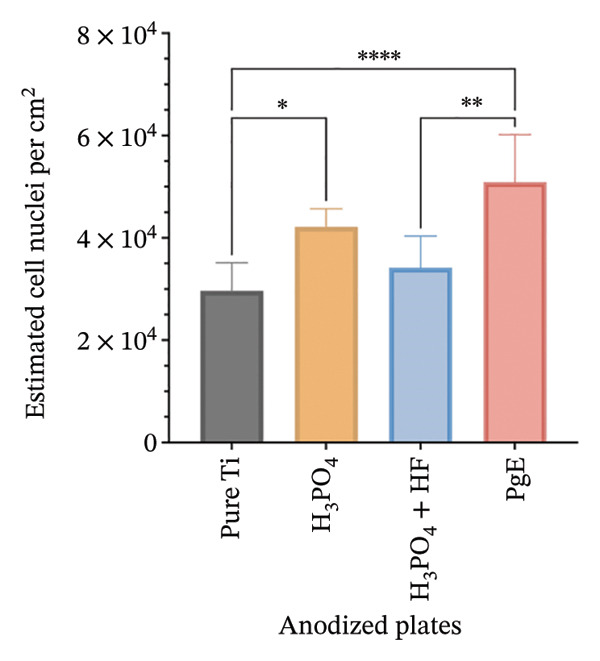
(c)

Furthermore, plates anodized with PgE showed a 35.7% higher number of nuclei compared to those treated with H_3_PO_4_ + HF. In Saos‐2 cells, plates anodized with H_3_PO_4_ resulted in a 42% increase in the number of nuclei compared to pure titanium, while the PgE induced a 71.6% increase. Additionally, when comparing the H_3_PO_4_ + HF treatment with the PgE anodized plates, the latter demonstrated a 32.9% higher number of nuclei.

The proliferation and adhesion results, which were used as parameters to evaluate osseointegration, showed that titanium anodization generally enhances biocompatibility, as evidenced by the behavior of NIH‐3T3 cells. This finding is further supported by studies with human gingival fibroblasts, which show improved cellular responses and suggest a positive influence on connective tissue regeneration compared to nonanodized titanium surfaces [36]. The embryo fibroblast‐like cells are known for their high proliferative capacity and key role in the early stages of tissue repair, as they produce the matrix that supports new tissue formation, assist in activating osteoblasts, and respond to signals that regulate bone growth and repair [37].

In Saos‐2 cells, a bone‐derived osteosarcoma cell line that exhibits slower proliferation, the results were more modest. Titanium anodized with H_3_PO_4_ + HF showed no significant improvement in cell adhesion compared to pure titanium. Surface analysis studies indicate that electrochemical treatment in phosphoric and hydrofluoric acid solutions promotes the formation of nanoscale pores. However, this condition results in a lower amount of phosphate adsorption on the surface [38], which may compromise the initial adhesion of cells that depend on phosphate for osteogenic growth and differentiation [36].

Plates anodized with PgE yielded results comparable to those obtained with H_3_PO_4_ in both cell lines. This similarity may be associated with the presence of phosphate in the PgE electrolyte. Phosphorus is an essential mineral that, in the form of inorganic phosphate (Pi), is required for the formation of cell membranes, DNA and RNA molecules, and energy metabolism [39]. Indeed, studies on odontoblast‐like cells have demonstrated that, at appropriate concentrations, H_3_PO_4_ stimulates proliferation and mineralization of mouse and rat undifferentiated dental pulp cells [37]. Consistent with these findings, MC3T3‐E1 osteoblasts cultured on H_3_PO_4_‐anodized titanium surfaces displayed increased phosphate incorporation into the oxide layer, leading to a 157% increase in surface roughness, improved wettability, enhanced cell adhesion, upregulation of osteogenic gene expression, and stronger biomechanical anchorage [24].

However, given that its effects closely parallel those of phosphoric acid, the comparable biological performance observed for PgE and H_3_PO_4_ may reflect analogous modifications in surface topography and hydrophilicity. Another possibility is that the secondary metabolites, such as flavonoids and phenolic acids, present in PgE exhibit antioxidant and cytoprotective effects, reducing oxidative stress and increasing glutathione levels [40]. Nevertheless, further investigations are required to elucidate the mechanisms through which PgE promotes cell proliferation and adhesion.

## 4. Conclusions

In this study, anodized titanium plates with H_3_PO_4_, H_3_PO_4_ + HF or PgE produce uniform oxide films. These anodized plates did not induce cytotoxicity and promoted enhanced cell adhesion and proliferation. Anodization markedly improved key indicators of osseointegration, yielding higher cell density on H_3_PO_4_ and PgE anodized titanium plates, as well as more extensive cell spreading and tighter intercellular contacts. In contrast, H_3_PO_4_ + HF anodized plates exhibited comparatively weaker cell adhesion, like pure titanium.

These findings indicate that the use of PgE as an alternative electrolyte on titanium plates exhibited no cytotoxicity while promoting cell adhesion and proliferation, representing a promising and innovative approach to metallic surface modification. However, in vivo studies are required to refine the understanding of osseointegration and to assess biochemical markers and inflammatory responses, to confirm its potential for clinical application.

## Author Contributions

According to the CRediT contributorship taxonomy, Felipe Gustavo Dias performed the roles of conceptualization, investigation, and writing–original draft. Gabriela Zimmermann Prado Rodrigues performed the roles of investigation, methodology, and supervision. Isadora Schell Frozza performed the role of methodology. Carlos Henrique Amaro da Silva performed the role of methodology. Fernando Dal Pont Morisso performed the roles of conceptualization, resources, and writing–review and editing. Cláudia Trindade Oliveira performed the roles of conceptualization, funding acquisition, methodology, project administration, and writing–review and editing. Günther Gehlen performed the roles of methodology, resources, supervision, and writing–review and editing. Ana Luiza Ziulkoski performed the roles of conceptualization, funding acquisition, methodology, project administration, resources, supervision, and writing–review and editing.

## Funding

The research was supported by Financiadora de Estudos e Projetos (Finep, Call for Proposals No. 0164/21–MCTI/FINEP/AT Advanced Materials and Strategic Minerals 2020) and Conselho Nacional de Desenvolvimento Científico e Tecnológico (CNPq) through the granting of master and undergraduate scholarships.

## Conflicts of Interest

The authors declare no conflicts of interest.

## Data Availability

The data that support the findings of this study are available from the corresponding author upon reasonable request.
